# Cooperative function of Fmp30, Mdm31, and Mdm32 in Ups1-independent cardiolipin accumulation in the yeast *Saccharomyces cerevisiae*

**DOI:** 10.1038/s41598-017-16661-2

**Published:** 2017-11-27

**Authors:** Non Miyata, Naoto Goda, Keiji Matsuo, Takeshi Hoketsu, Osamu Kuge

**Affiliations:** Department of Chemistry, Faculty of Science, Kyushu University, Fukuoka, 819-0395 Japan

## Abstract

Cardiolipin (CL) is synthesized from phosphatidic acid (PA) through a series of enzymatic reactions occurring at the mitochondrial inner membrane (MIM). Ups1-Mdm35 mediates PA transfer from the mitochondrial outer membrane (MOM) to the MIM in the yeast *Saccharomyces cerevisiae*. Deletion of *UPS1* leads to a ~80% decrease in the cellular CL level. However, the CL accumulation in *ups1∆* cells is enhanced by the depletion of Ups2, which forms a protein complex with Mdm35 and mediates phosphatidylserine (PS) transfer from the MOM to the MIM for phosphatidylethanolamine (PE) synthesis by a PS decarboxylase, Psd1. In this study, we found that the accumulation of CL in *ups1∆* cells was enhanced by deletion of not only *UPS2*, but also *PSD1* and *CHO1* encoding a PS synthase, suggesting that low PE levels in mitochondria were relevant to the enhancement of CL accumulation in *ups1∆* cells. Furthermore, the *Ups1*-independent and low-level PE-enhanced CL accumulation was shown to depend on the functions of *FMP30*, *MDM31*, and *MDM32*. In addition, the physical interactions of Fmp30 with Mdm31 and Mdm32 were revealed. Thus, when the mitochondrial PE level is reduced, Fmp30, Mdm31, and Mdm32 seem to function cooperatively for the accumulation of CL in a *UPS1*-independent manner.

## Introduction

Cardiolipin (CL) is a unique dimeric glycerolphospholipid localized almost exclusively to mitochondria in mammalian cells and the yeast *Saccharomyces cerevisiae*. CL plays critical roles in mitochondrial functions, such as oxidative phosphorylation, and regulation of apoptosis and mitophagy^1–3^, and involved in the biogenesis of mitochondria through the regulation of protein import^4–7^ into and fusion^8^ of mitochondria. This phospholipid exhibits a propensity for the formation of non-bilayer, inverted hexagonal (H_II_) phase structures^9^, and thus seems to engage in the formation of local non-bilayer structures within mitochondrial membranes, which may be involved in membrane dynamics, including membrane assembly, transmembrane movement of proteins, and dynamic formation of membrane contact sites.

**Figure 1:**
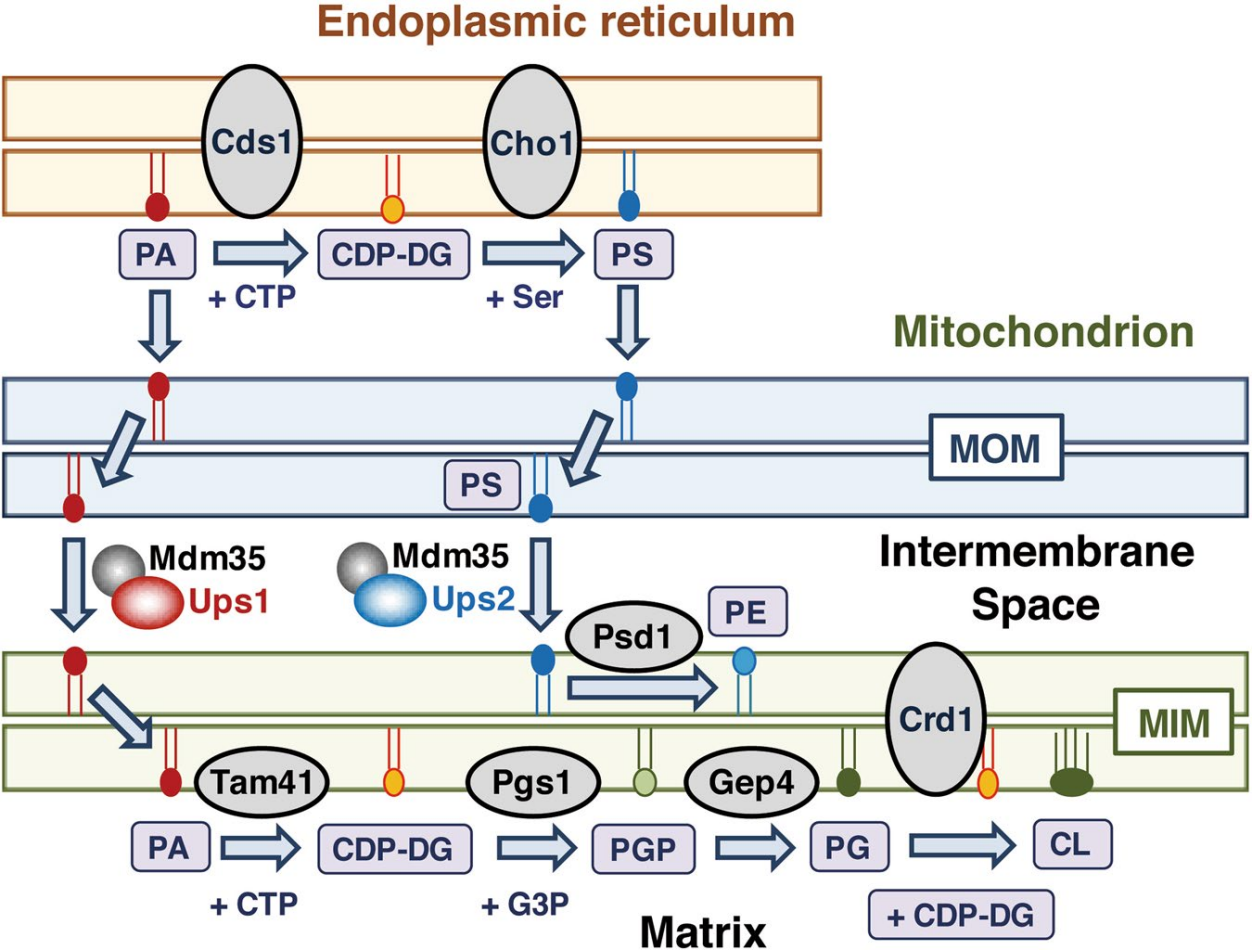
Mitochondrial synthesis of cardiolipin and phosphatidylethanolamine in yeast. CDP-DG, CDP-diacylglycerol; CL, cardiolipin; G3P, glycerol 3-phosphate; MIM, mitochondrial inner membrane; MOM, mitochondrial outer membrane; PA, phosphatidic acid; PE, phosphatidylethanolamine; PG, phosphatidylglycerol; PGP, phosphatidylglycerol phosphate; PS, phosphatidylserine.

Mitochondria of the yeast *S. cerevisiae* contain a set of enzymes required for CL biosynthesis from phosphatidic acid (PA), a common intermediate for phospholipid biosynthesis. The CL biosynthetic pathway in the yeast was shown in Fig. 1. Tam41 catalyzes the first step of the pathway, namely, the synthesis of CDP-diacylglycerol (CDP-DG) from PA and CTP^10^. The second step is the formation of phosphatidylglycerol phosphate (PGP) from CDP-DG and glycerol 3-phosphate, which is catalyzed by PGP synthase (Pgs1)^11,12^. The third step is dephosphorylation of PGP by Gep4, resulting in the formation of phosphatidylglycerol (PG)^13^. In the final step, a cardiolipin synthase, Crd1, catalyzes the CL formation from PG and CDP-DG^14–16^. These enzymes of the CL biosynthetic pathway are associated with the matrix face of or inserted into the mitochondrial inner membrane (MIM) (Fig. 1). Mitochondria are incapable of synthesizing PA *de novo*, and therefore PA produced in the extramitochondrial space such as the endoplasmic reticulum and lipid droplets should be transported to the MIM for CL biosynthesis. So far, Ups1-Mdm35, a protein complex localized to the mitochondrial intermembrane space (IMS), has been shown to mediate PA transfer from the mitochondrial outer membrane (MOM) to the MIM^17–20^.

The Ups1-Mdm35-mediated PA transfer is an important process for CL biosynthesis, because deletion of *UPS1* (*ups1∆*) leads to a ~80% decrease in the cellular CL level^21,22^. However, CL synthesis occurs even in yeast cells lacking Ups1 and the CL synthesis in *ups1∆* cells is strikingly enhanced by depletion of Ups2^21^, which forms a protein complex with Mdm35^23,24^ and mediates phosphatidylserine (PS) transfer from the MOM to the MIM for phosphatidylethanolamine (PE) synthesis by a PS decarboxylase, Psd1^25,26^. Therefore, a Ups1-independent PA transfer pathway to the MIM for CL biosynthesis exists in the yeast; elucidation of that pathway is important for clarification of the whole picture of CL metabolism. In this study, we show that the CL accumulation through the Ups1-independent CL biosynthetic pathway is enhanced by deletion of not only *UPS2*, but also *PSD1* and *CHO1* encoding a PS synthase, both of which are required for mitochondrial PE synthesis, suggesting that low PE levels are relevant to the enhancement of CL accumulation through the Ups1-independent pathway, and that the Ups1-independent and low-level PE-enhanced CL accumulation depends on the functions of *FMP30*, *MDM31*, and *MDM32*, which have been shown to be involved in CL metabolism^22,27,28^.

## Results

### The PE level is relevant to the accumulation of CL in and growth of *ups1∆* cells

**Figure 2:**
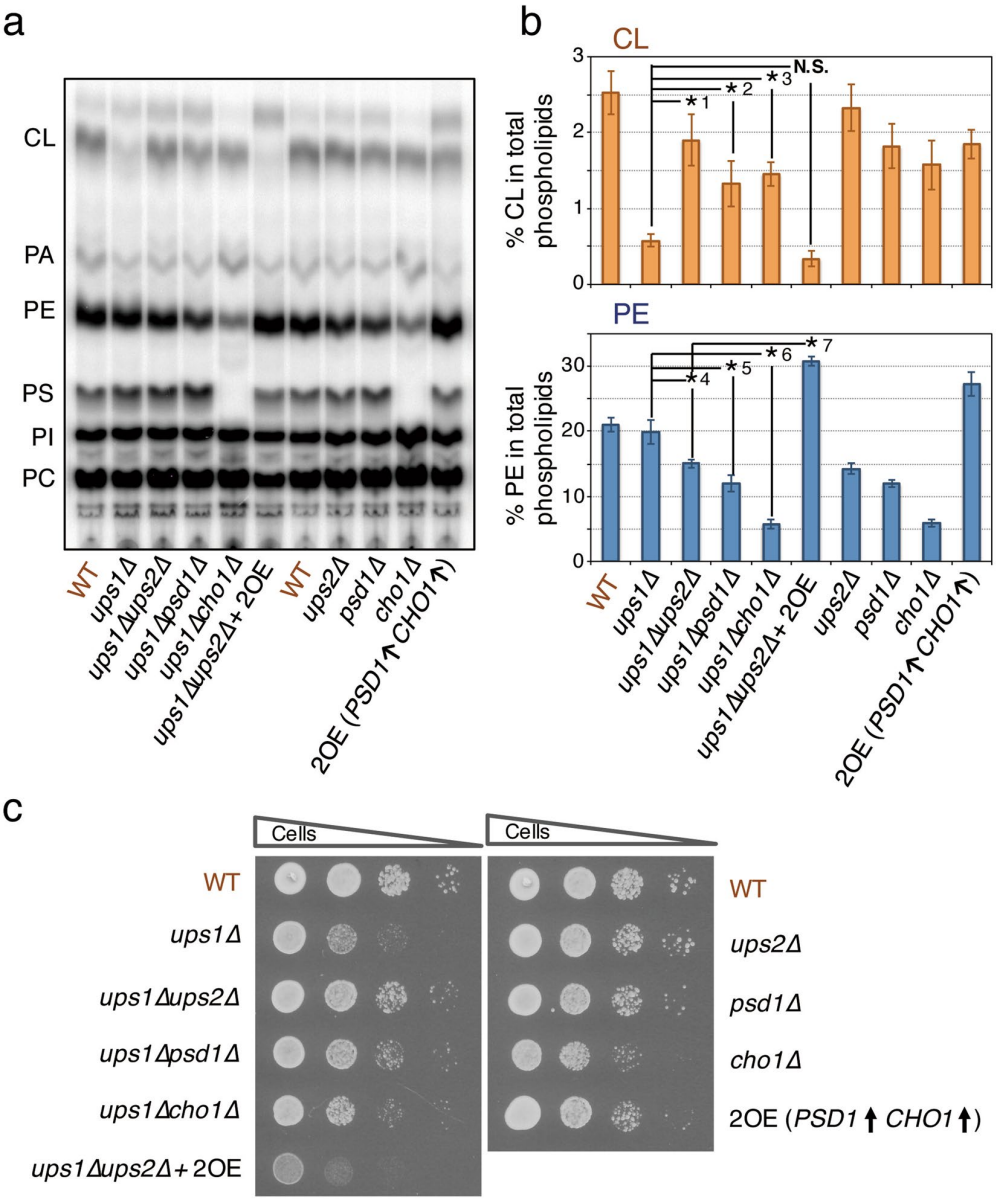
The PE level is relevant to the accumulation of CL in and growth of *ups1∆* cells. (**a** and **b**) Yeast cells, as indicated, were grown at 30 °C to saturation in YPAD medium. The cells were then diluted to an OD_600_ of 0.05 in 500 µl of YPAD containing [^32^P]Pi (10 µCi/ml) and cultured at 30 °C for 24 hours. Total cellular phospholipids were extracted, separated by TLC and then analyzed with an imaging analyzer. (**a**) Typical chromatogram on phospholipid analysis by TLC. PI, phosphatidylinositol; PC, phosphatidylcholine. (**b**) The percentages of CL and PE relative to total major phospholipids (CL, PA, PE, PS, PI, and PC). Values are means ± SD (*n* = 3~15). (**c**) Growth of various mutant cells. Yeast cells, as indicated, were spotted onto YPAD plates in ten-fold serial dilutions starting with a density of 0.7 OD_600_ units/ml, and then incubated at 30 °C for 24 hours. *1, *p* = 0.015; *2, *p* = 0.040; *3, *p* = 0.010; *4, *p* = 0.022; *5, *p* = 0.0022; *6, *p* = 0.0027; *7, *p* = 0.0095. N.S., not significant.

The defect in CL accumulation in *ups1∆* cells has been shown to be suppressed by deletion of *UPS2* ^21^, which leads to a decrease in the PE level in mitochondria^21,22,25,28^. Therefore, we examined the effect of deletion of *PSD1* and *CHO1*, both of which are required for the mitochondrial PE synthesis, on the accumulation of CL in *ups1∆* cells. Figure 2a shows thin layer chromatography (TLC) analyses of the total cellular [^32^P]phospholipids of various mutant cells metabolically labeled for 24 hours with [^32^P]Pi. The CL level in *ups1∆* cells was about 20% of that in wild-type cells, and increased to about 75% of that in wild-type cells after introduction of the *ups2∆* mutation (Fig. 2a,b), this being consistent with previous studies^21^. To our surprise, deletion of *PSD1* and *CHO1* as well as *UPS2* in *ups1∆* cells resulted in an increase in the CL level, as shown in Fig. 2a,b. The CL levels in *ups1∆psd1∆* and *ups1∆cho1∆* double mutant cells, respectively, were 52 and 58% of that in wild-type cells and slightly lower than that in *psd1∆* and *cho1∆* single mutant cells (Fig. 2a,b). The PE levels in *ups1∆ups2∆*, *ups1∆psd1∆*, and *ups1∆cho1∆* double mutant cells, respectively, were about 70, 60, and 25% of that in wild-type cells, and similar to those in *ups2∆*, *psd1∆*, and *cho1∆* single mutant cells (Fig. 2a,b). Furthermore, concomitant overexpression of *PSD1* and *CHO1* (2OE or *PSD1*↑*CHO1*↑) under the control of a strong glyceraldehyde-3-phosphate dehydrogenase (GPD) promoter in *ups1∆ups2∆* cells increased the PE level and abolished the enhancement of CL accumulation in *ups1∆* cells caused by the *ups2∆* mutation (Fig. 2a,b).

We also examined the growth rates of the various mutants used for the above experiments by means of a spot test, and found correlation of the cellular CL levels and growth rates, as shown in Fig. 1c. These results suggest that a decrease in PE to under the threshold level enhances CL accumulation in *ups1∆* cells and suppresses the growth defect of *ups1∆* cells.

### *FMP30* is required for the *UPS1*-independent and low-level PE-enhanced CL accumulation

**Figure 3:**
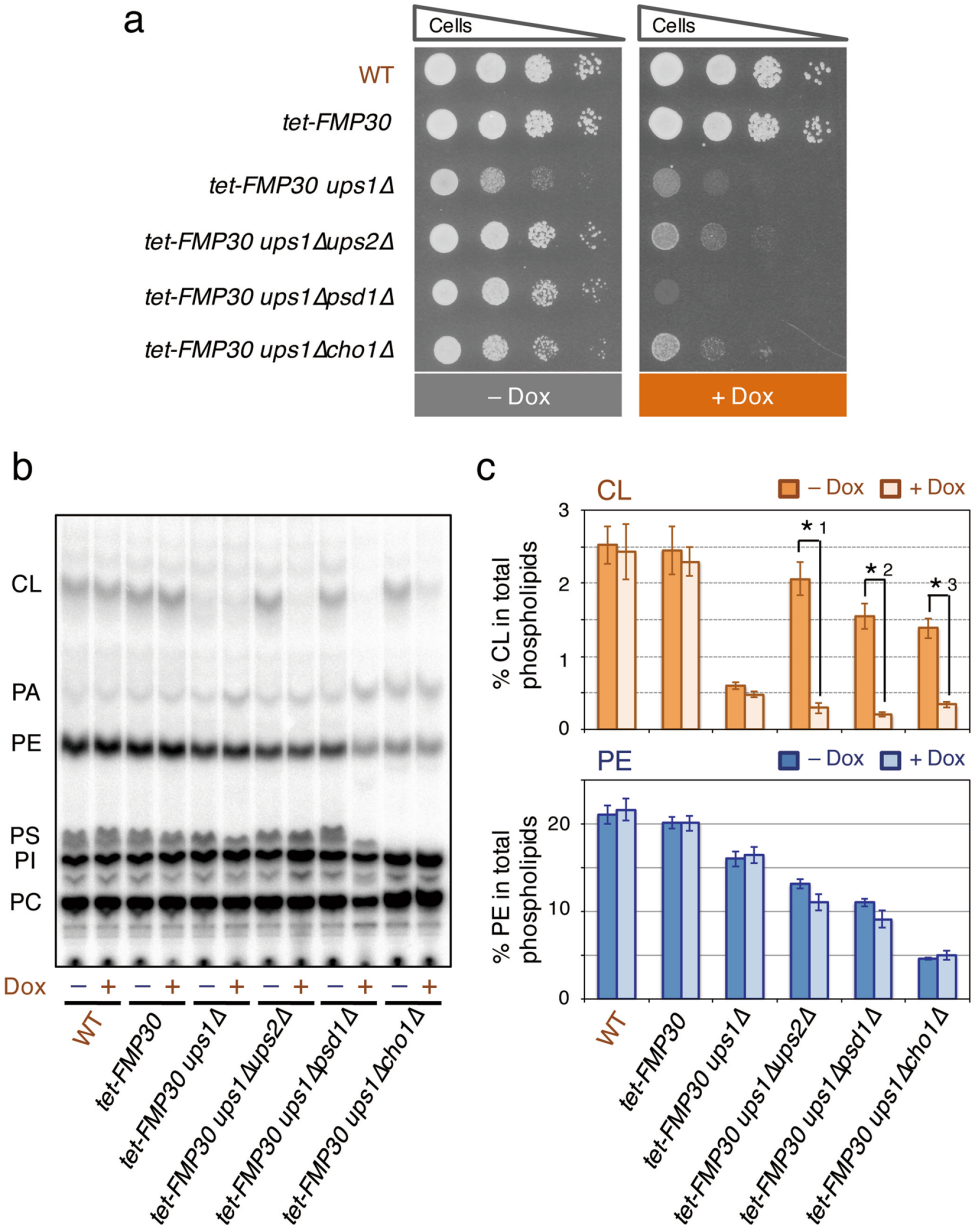
*FMP30* is required for the *UPS1*-independent and low-level PE-enhanced accumulation of CL. (**a**) Growth of various mutant cells. Yeast cells, as indicated, were grown at 30 °C to saturation in YPAD medium with or without 10 μg/ml Dox, as indicated. The yeast cells were then spotted onto YPAD plates with or without 10 μg/ml Dox, as indicated, in ten-fold serial dilutions starting with a density of 0.7 OD_600_ units/ml, and then incubated at 30 °C for 24 hours. (**b** and **c**) Yeast cells, as indicated, were grown at 30 °C to saturation in YPAD medium with or without 10 μg/ml Dox, as indicated. The cells were then diluted to an OD_600_ of 0.05 in 500 µl of YPAD containing [^32^P]Pi (10 µCi/ml), supplemented with or without 10 μg/ml Dox and cultured at 30 °C for 24 hours. Total cellular phospholipids were extracted, separated by TLC, and then analyzed with an imaging analyzer. (**b**) Typical chromatogram on phospholipid analysis by TLC. (**c**) The percentages of CL and PE relative to total major phospholipids (CL, PA, PE, PS, PI, and PC). Values are means ± SD (*n* = 3~15). *1, *p* = 0.035; *2, *p* = 0.0044; *3, *p* = 0.0050.

*FMP30* encodes a mitochondrial inner membrane protein having a large domain exposed to the intermembrane space, which exhibits strong homology with mammalian N-acylPE (NAPE)-specific phospholipase Ds (NAPE-PLDs)^27,29^. We have shown that *FMP30* is required for the maintenance of a normal CL level in *psd1∆* cells, and that deletion of *FMP30* is synthetically lethal with the *ups1∆* mutation^27^. Therefore, we decided to examine whether *FMP30* was involved in the *UPS1*-independent and low-level PE-enhanced CL accumulation. For this purpose we constructed yeast strains carrying the *FMP30* gene under the control of a tetracycline-regulatable TET_off_ promoter^30^ (*tet-FMP30*) with or without various mutations including *ups1∆*. Wild-type and *tet-FMP30* cells were able to grow well equally in the presence or absence of a tetracycline analogue, doxycycline (Dox) (Fig. 3a). However, *tet-FMP30 ups1∆* cells showed a partial growth defect in the absence of Dox and a further strong growth defect in the presence of Dox, as expected (Fig. 3a), indicating that Dox depressed *tet-FMP30* gene expression.

Figure 3b shows TLC analysis of the total cellular [^32^P]phospholipids of wild-type and various *tet-FMP30*-carrying mutant cells metabolically labeled with [^32^P]Pi for 24 hours in the presence or absence of Dox. The CL and PE levels in *tet-FMP30* cells were not significantly affected by the addition of Dox to the growth medium, as was the case with those in wild-type cells, and similar to those in wild-type cells cultivated with or without Dox (Fig. 3b,c). The CL level in *tet-FMP30 ups1∆* cells was about 20% of that in *tet-FMP30* cells in the medium without Dox, which is similar to that in *ups1∆* cells with the native *FMP30* gene (Figs 2b and 3c). Although the addition of Dox to the medium significantly affected the growth of *tet-FMP30 ups1∆* cells (Fig. 3a), the Dox addition did not lead to a significant further decrease in the CL level in *tet-FMP30 ups1∆* cells (Fig. 3b,c), implying that Fmp30 was not involved in the accumulation of the residual amount of CL in *ups1∆* cells. In contrast, the *UPS1*-dependent and low-level PE-enhanced CL accumulation, namely, the CL accumulation in *ups1∆ups2∆*, *ups1∆psd1∆*, and *ups1∆cho1∆* double mutant cells, were strikingly reduced by repression of *tet-FMP30* expression by Dox (Fig. 3b,c). In addition, the growth of *ups1∆ups2∆*, *ups1∆psd1∆*, and *ups1∆cho1∆* cells carrying *tet-FMP30* was significantly impaired by the addition of Dox to the medium, as shown in Fig. 3a. These results suggest that *FMP30* is required for the *UPS1*-independent and low-level PE-enhanced accumulation of CL.

**Figure 4:**
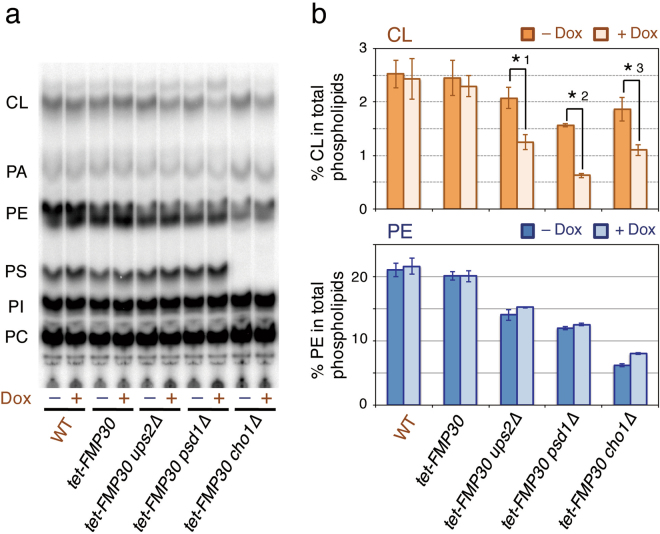
*ups2∆*, *psd1∆*, and *cho1∆* mutations change the *FMP30-*dependent pathway from a minor to a major one for accumulation of CL. (**a** and **b**) See the legend to Fig. 3b,c. (**a**) Typical chromatogram on phospholipid analysis by TLC. (**b**) The percentages of CL and PE relative to total major phospholipids (CL, PA, PE, PS, PI, and PC). Values are means ± SD (*n* = 3~15). *1, *p* = 0.0022; *2, *p* = 0.0019; *3, *p* = 0.013.

As control experiments, we subjected *tet-FMP30* cells having the *ups2∆*, *psd1∆*, or *cho1∆* mutation, but not the *ups1∆* mutation to phospholipid analyses, obtaining interesting results. Although the CL level in *tet-FMP30* cells was not significantly affected by the addition of Dox to the medium, those in *ups2∆*, *psd1∆*, and *cho1∆* cells carrying *tet-FMP30* were decreased by about 40, 60, and 40%, respectively, by the Dox addition (Fig. 4b). These results implied that the *ups2∆*, *psd1∆*, and *cho1∆* mutations changed the *FMP30*-dependent pathway for CL accumulation from a minor one to a major one.

### *MDM31* and *MDM32* are required for the *UPS1*-independent and low-level PE-enhanced CL accumulation

**Figure 5:**
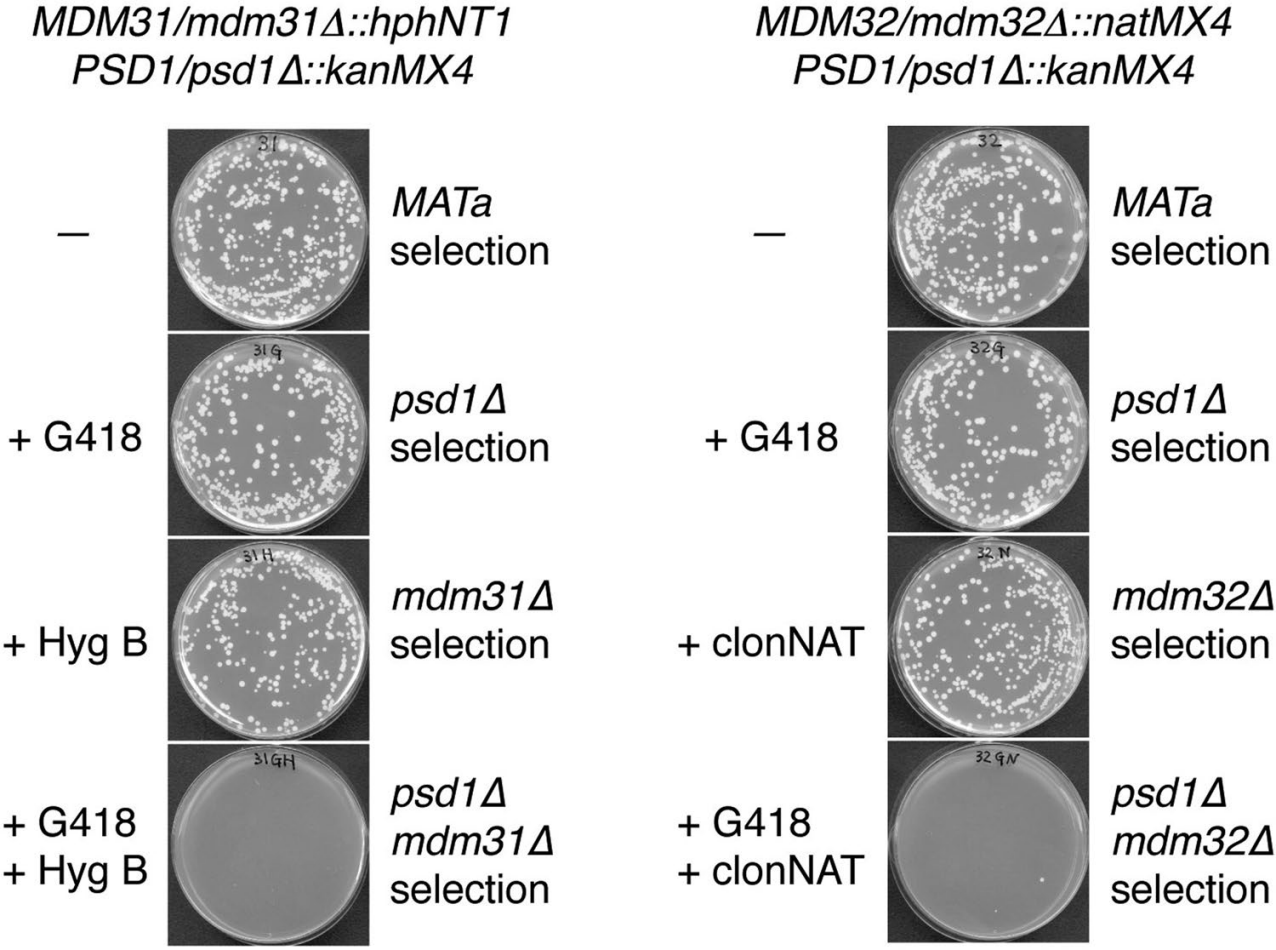
Deletion of *MDM31* and *MDM32* are synthetically lethal with deletion of *PSD1*. A yeast strain, *psd1∆* α (*MAT*α *psd1∆::kanMX4 can1∆::STE2pr-Sp_his5*), was mated with another strain, *mdm31∆* (*MAT*a *mdm31∆::hphNT1 CAN1* ^*WT*^) or mdm32∆ (*MAT*a *mdm32∆::natNT2 CAN1* ^*WT*^). The resulting heterozygous diploid strains ((*PSD1/psd1∆::kanMX4 MDM31/mdm31∆::hphNT1 CAN1/can1∆::STE2pr-Sp_his5*) and (*PSD1/psd1∆::kanMX4 MDM31/mdm31∆::hphNT1 CAN1/can1∆::STE2pr-Sp_his5*)) were allowed to sporulate, plated on the various selection media indicated, and then incubated at 30 °C for 3 days. All the media contained Canavanine but not histidine for the selection of MATa haploid strains.

**Figure 6:**
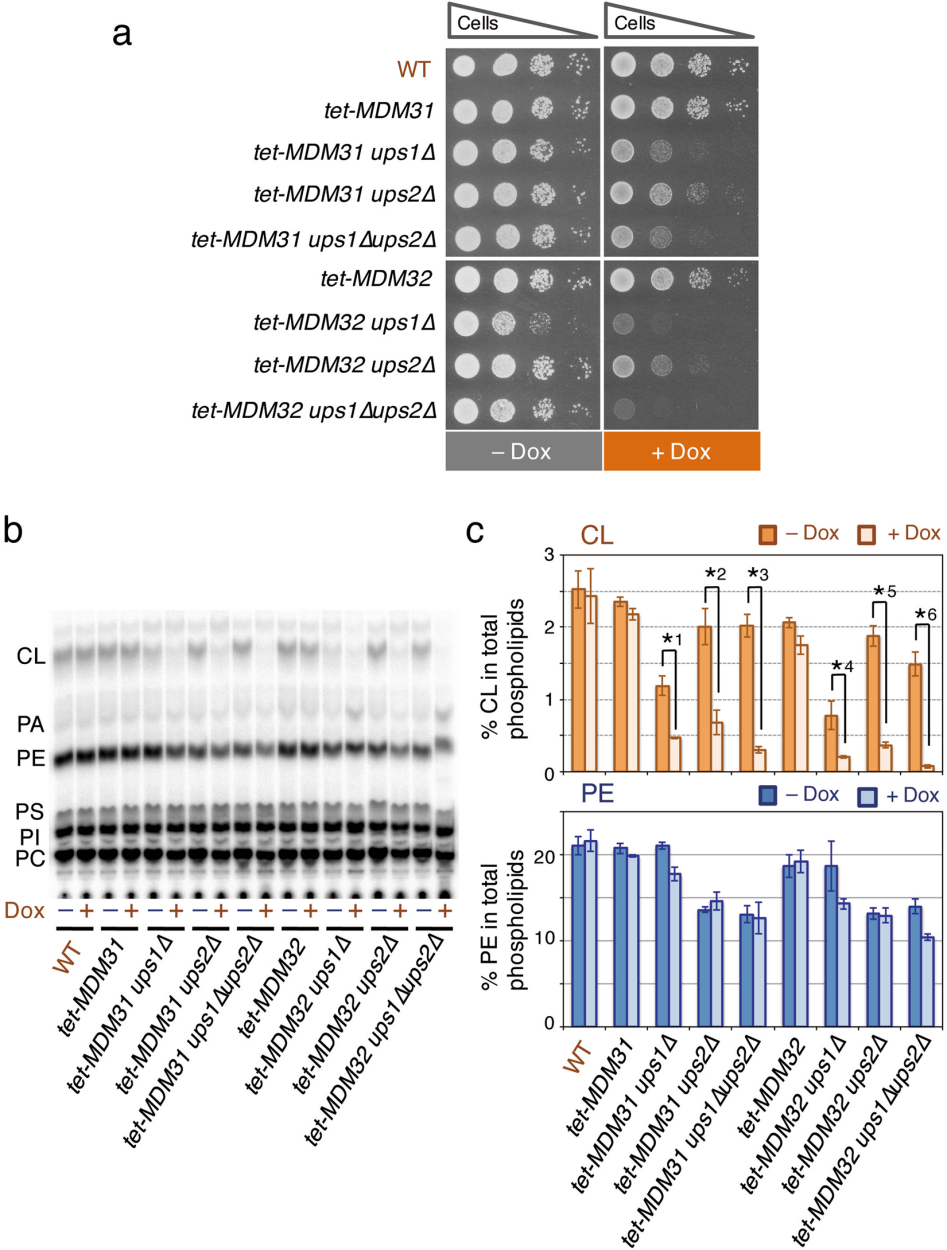
*MDM31* and *MDM32* are required for the *UPS1*-independent and low-level PE-enhanced accumulation of CL. (**a**) Growth of various mutant cells. See the legend to Fig. 3a. (**b** and **c**) See the legend to Fig. 3b,c. (**b**) Typical chromatogram on phospholipid analysis by TLC. (**c**) The percentages of CL and PE relative to total major phospholipids (CL, PA, PE, PS, PI, and PC). Values are means ± SD (*n* = 3~15). *1, *p* = 0.0092; *2, *p* = 0.0012; *3, *p* = 0.0015; *4, *p* = 0.035; *5, *p* = 0.0027; *6, *p* = 0.0057.

We have identified *FMP30* as a gene whose deletion in *psd1∆* cells causes a severe growth defect^27^. During the course of such a study of genetic interactions, we found also that deletion of *MDM31* is synthetically lethal with the *psd1∆* mutation^27^. Mdm31 and its homolog Mdm32 are mitochondrial inner membrane proteins with two membrane-spanning regions at the C-terminus and near the N-terminus, respectively, and a middle region exposed to the intermembrane space, and have been shown to be involved in CL metabolism^22,28^. As shown in Fig. 5, deletion of *MDM32* as well as *MDM31* was shown to be synthetically lethal with the *psd1∆* mutation. Therefore, because of their similarities to *FMP30* in submitochondrial localization and genetic interaction with *PSD1*, *MDM31* and *MDM32* might be involved in the *UPS1*-independent and low-level PE-enhanced accumulation of CL. In fact, overexpression of *MDM31* partially suppresses the defect in growth of and CL accumulation in *ups1∆* cells^28^. To address the functions of *MDM31* and *MDM32* in *ups1∆* cells, we constructed yeast strains carrying the *MDM31* or *MDM32* gene under the control of a tetracycline-regulatable TET_off_ promoter (*tet-MDM31* or *tet-MDM32*) with or without the *ups1∆* and/or *ups2∆* mutation(s). Wild-type, *tet-MDM31*, and *tet-MDM32* cells were able to grow well equally in the presence or absence of Dox (Fig. 6a). However, the growth of *tet-MDM31* and *tet-MDM32* cells carrying the *ups1∆* or *ups2∆* mutation, and both the *ups1∆* and *ups2∆* mutations was impaired by the addition of Dox to the medium (Fig. 6a).

Next, we examined the CL levels in these mutant cells cultivated in the presence or absence of Dox by metabolic labeling with [^32^P]Pi for 24 hours, followed by TLC analysis. The CL level in *tet-MDM31* cells was about 95% of that in wild-type cells in the medium without Dox, and decreased by 7% on the addition of Dox to the medium (Fig. 6b,c). The CL level in *tet-MDM31 ups1∆* cells cultivated without Dox was 48% of that in wild-type cells and about 2.5-fold that in *ups1∆* cells (Figs 2a,b and 6b,c). This elevation of the CL level in *ups1∆* cells on replacement of the native *MDM31* promoter with the TET_off_ promoter might have resulted from an increase in the expression level of the gene, because overexpression of *MDM31* had been shown to partially suppress the defect in CL accumulation in *ups1∆* cells^28^. The addition of Dox to the medium reduced the CL level in *tet-MDM31 ups1∆* cells to a level similar to that in *ups1∆* cells (Figs 2a,b and 6b,c), indicating that *MDM31* is involved in the *UPS1*-independent CL accumulation. However, because there was no significant difference between the CL levels in *ups1∆* cells and *tet-MDM31 ups1∆* cells cultivated with Dox (Figs 2a,b and 6b,c), *MDM31* might not be involved in the accumulation of the residual amount of CL in *ups1∆* cells. In the medium without Dox, the CL level in *tet-MDM31 ups1∆ ups2∆* cells was about 80% of that in wild-type cells and about 1.7-fold that in *tet-MDM31 ups1∆* cells (Fig. 6b,c), indicating that the *UPS1*-independent and low-level PE-enhanced CL accumulation had occurred. In contrast to the CL level in *tet-MDM31* cells, that in *tet-MDM31 ups1∆ ups2∆* cells was strikingly reduced by depression of *tet-MDM31* expression by Dox, as shown in Fig. 6b,c, suggesting that *MDM31* was required for the *UPS1*-independent and low-level PE-enhanced CL accumulation.

As shown in Fig. 6b,c, the CL levels in *tet-MDM32*, *tet-MDM32 ups1∆*, and *tet-MDM32 ups1∆ups2∆* cells cultivated with or without Dox, respectively, were similar to those in *tet-MDM31*, *tet-MDM31 ups1∆*, and *tet-MDM31 ups1∆ups2∆* cells cultivated with or without Dox. It is therefore likely that *MDM32* is also required for the *UPS1*-independent and low-level PE-enhanced CL accumulation.

Although the accumulation of CL in and growth of *tet-MDM31* and *tet-MDM32* cells were not largely affected by the addition of Dox to the medium, those of *ups2∆* cells carrying *tet-MDM31* or *tet-MDM32* were remarkably impaired by the Dox addition (Fig. 6b,c). These results implied that the *ups2∆* mutation changed the main CL biosynthetic pathway from an *MDM31*- and *MDM32*-independent pathway to an *MDM31*- and *MDM32*-dependent pathway.

### Physical interaction of Fmp30 with Mdm31 and Mdm32

**Figure 7:**
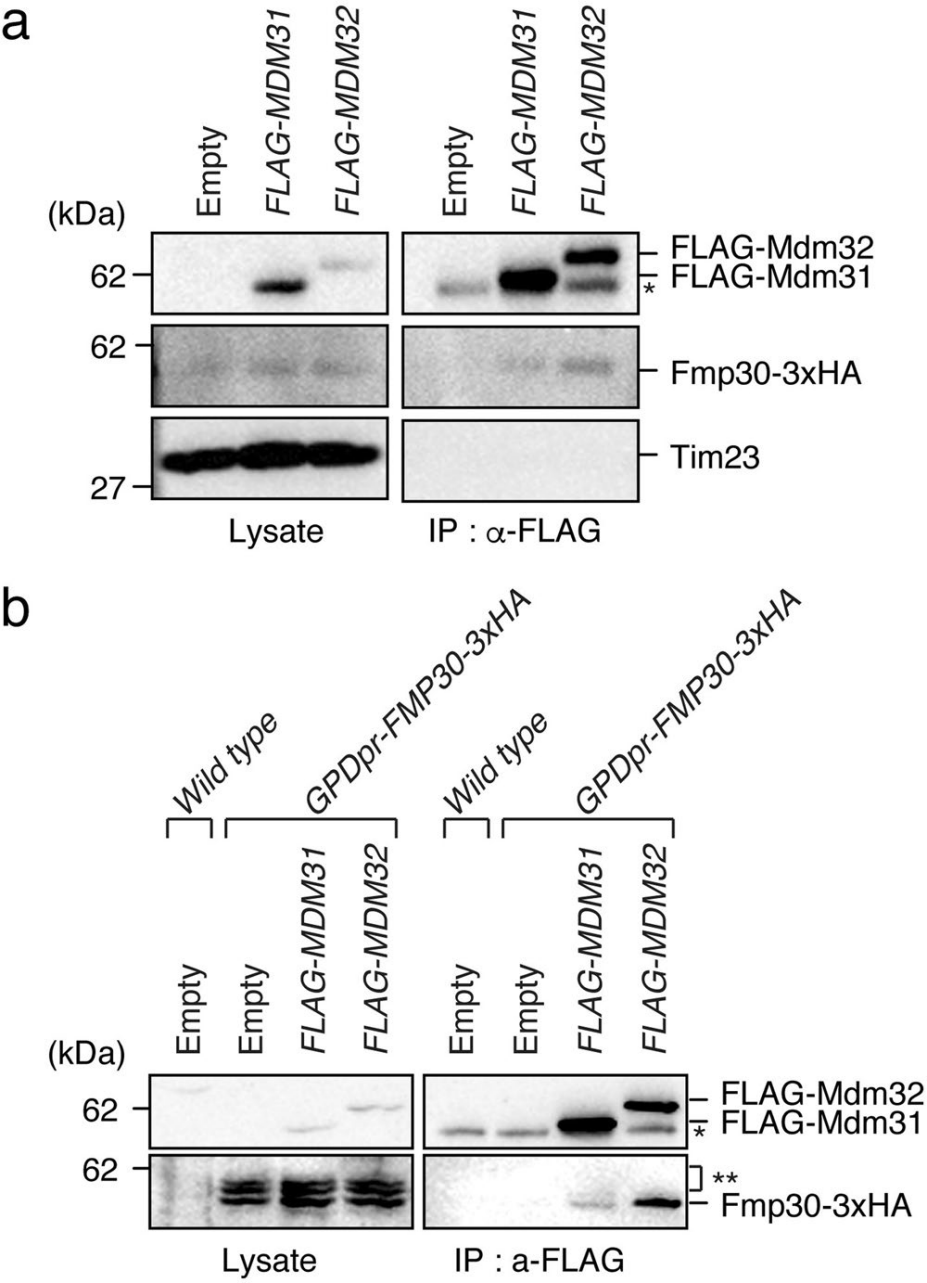
Fmp30 physically interacts with Mdm31 and Mdm32. (**a**) Mitochondria (2 mg) from the yeast cells expressing genomically*-3xHA*-tagged *FMP30*, and carrying the plasmid pRS426 (Empty), pRS426-*FLAG-MDM31* (*FLAG-MDM31*), or pRS426-*FLAG-MDM32* (*FLAG-MDM32*) were solubilized with 1% digitonin and then subjected to immunoprecipitation with anti-FLAG agarose beads. The immunoprecipitates were eluted from the beads with 2% SDS and then analyzed by Western blotting with anti-HA, anti-FLAG, and anti-Tim23 antibodies. The asterisk indicates the IgG from the anti-FLAG agarose, which migrates similarly to FLAG-Mdm31. (**b**) Mitochondria (2 mg) from wild-type or the yeast cells expressing genomically-*3xHA-tagged FMP30* under GPD promotor and carrying the plasmid pRS424 (Empty), pRS424-*FLAG-Mdm31* (*FLAG-MDM31*), or pRS424-*FLAG-Mdm32* (*FLAG-MDM32*) were analyzed as described in (**a**). *indidates the IgG from the anti-FLAG agarose, which migrates similarly to FLAG-Mdm31. **indicates the potential unprocessed/precursor forms of Fmp30-3xHA, which may accumulate due to the overproduction of Fmp30. Note that the fastest-migrating form of Fmp30-3xHA, which appeared to be mature form, was co-precipitated with FLAG-Mdm31 and FLAG-Mdm32. Multiple exposures of full-length blots are presented in Supplementary Figure S1.

We showed that depletion of any one of the factors Fmp30, Mdm31, and Mdm32 almost completely prevented CL synthesis under the mitochondrial-PE-reduced and Ups1-defective conditions (Figs 3 and 6), suggesting that these three factors act in the same pathway. To obtain further evidence for this, we performed immunoprecipitation experiments. Mitochondria were isolated from the yeast cells expressing genomically*-3xHA*-tagged *FMP30* and carrying the plasmid pRS426, pRS426-*FLAG-MDM31*, or pRS426-*FLAG-MDM32*, and then subjected to immunoprecipitation with anti-FLAG agarose beads. As shown in Fig. 7a and S1, Fmp30-3xHA was co-immunoprecipitated with both FLAG-Mdm31 and FLAG-Mdm32, although Fmp30-3xHA was detected more strongly in the immunoprecipitate fraction of FLAG-Mdm32 than that of FLAG-Mdm31. In contrast, another inner membrane protein, Tim23, was detected in neither the immunoprecipitate fraction of FLAG-Mdm31 nor that of FLAG-Mdm32. To confirm the interactions of Fmp30 with Mdm31 and Mdm32, we performed immunoprecipitation experiments using the yeast cells overproducing Fmp30-HA under the control of a strong glyceraldehyde-3-phosphate dehydrogenase (GPD) promoter and carrying the plasmid encoding FLAG-Mdm31 or FLAG-Mdm32. As shown in Fig. 7b and S1, bands of Fmp30-3xHA were clearly detected in the immunoprecipitate fraction of FLAG-Mdm31 and that of FLAG-Mdm32. Thus, the immunoprecipitation experiments showed that Fmp30 physically interacted with both Mdm31 and Mdm32. These results, taken together, indicate that Fmp30, Mdm31 and Mdm32 cooperatively act in the same pathway, which is essential for CL synthesis under the mitochondrial-PE-reduced and Ups1-defective conditions.

## Discussion

CL and PE levels in and growth rates on YPAD medium of various mutant cells used in this study are summarized in Supplementary Table S3. In the present study, the enhancement of CL accumulation by *ups2∆* in *ups1∆* cells was suggested to be relevant to the decrease in the PE level by the finding that deletion of not only *UPS2* but also *PSD1* and *CHO1*, both of which are required for mitochondrial PE synthesis, suppressed the defect in CL accumulation in *ups1∆* cells (Fig. 2a,b), and that elevation of the PE level on concomitant overexpression of *PSD1* and *CHO1* (*PSD1*↑*CHO1*↑) in *ups1∆ ups2∆* cells abolished the increase in the CL level in *ups1∆* cells caused by the *ups2∆* mutation (Fig. 2a,b). How does a decrease in the PE level enhance the accumulation of CL in *ups1∆* cells? A previous study indicated that decreased PE levels have beneficial effects for maintenance of mitochondrial functions and morphology in yeast cells with a defect in the mitochondrial contact site and cristae organizing system (MICOS)^26^. Although deletion of *MIC10* or *MIC60*, both of which encode a core subunit of MICOS, leads to a partial defect in respiration growth and enlarged mitochondria with abnormally stacked multilamellar cristae observed on electron microscopy, deletion of *UPS2* in *mic10∆* and *mic60∆* cells improves respiration growth and restores the mitochondrial architecture with a normal cristae morphology^26^. In addition, deletion of *PSD1* in MICOS-deficient cells significantly improves respiratory growth^26^, though loss of Psd1 in wild-type cells reduces the PE level and impairs respiratory growth. Furthermore, deletion of *UPS2* improves cell growth and CL levels in yeast cells lacking the ER-mitochondria encounter structure (ERMES) complex^28^, which connects mitochondria to the ER and is suggested to facilitate phospholipid exchange between these two organelles^31,32^. On the other hand, it has been shown that the cristae morphology is disturbed upon inhibition of CL synthesis by the *tam41∆* or *pgs1∆* mutation but restored when PA transfer to the MIM by Ups1-Mdm35 is impaired^17^. These observations indicate that moderate alterations in lipid composition can have dramatic effects on mitochondrial functions and morphology. Thus, a decrease in the PE level in *ups1∆* cells might have a large effect on mitochondrial morphology, such as the number and extent of contact sites, and therefore enhance *UPS1*-independent PA supply to the MIM via contact sites for CL synthesis. Alternatively, a decrease in the PE level might nullify the inhibitory effect(s) of PE on Fmp30, Mdm31, and/or Mdm32, which are required for the *UPS1*-independent and low-level PE-enhanced CL accumulation (See below).

Although deletion of *UPS2*, *PSD1*, or *CHO1* restored the CL level in *ups1∆* cells, depression of *tet-FMP30* expression by Dox in *ups1∆ups2∆*, *ups1∆psd1∆*, and *ups1∆cho1∆* cells decreased the CL level to about 10% of that in wild-type cells (Fig. 3b,c). Similarly, depression of the *tet-MDM31* and *tet-MDM32* expression by Dox in *ups1∆ups2∆* cells decreased the CL level to about 10% or less of that in wild-type cells (Fig. 6b,c). These results suggest that all three genes, *FMP30*, *MDM31*, and *MDM32*, are essential for the *UPS1*-independent and low-level PE-enhanced CL accumulation, and therefore this CL accumulation is carried out through a single pathway, in which the three genes function cooperatively. Physical interactions of Fmp30 with Mdm31 and Mdm32 (Fig. 7), and positive genetic interactions of *FMP30* with *MDM31* and *MDM32* ^33^ support their cooperative functions in the same pathway. We hereafter call the *UPS1*-independent low-level PE-enhanced pathway the “FMM (Fmp30-, Mdm31, and Mdm32)-dependent” pathway.

In the wild-type cells, the *UPS1*-dependent pathway for CL synthesis appeared to be the main pathway for the CL accumulation, because deletion of *UPS1* led to an about 80% decrease in the CL level (Fig. 2a,b). However, depression of the *tet-FMP30* expression by Dox in *ups2∆*, *psd1∆*, and *cho1∆* cells with the wild-type allele of *UPS1*, respectively, led to about 40, 60, and 40% decreases in the CL level (Fig. 4a,b). Furthermore, repression of the *tet-MDM31* and *tet-MDM32* expression by Dox in *ups2∆* cells with the wild-type allele of *UPS1*, respectively, led to about 75 and 80% decreases in the CL level. These results suggest that in yeast cells with decreased mitochondrial PE levels, irrespective whether they carry a null (*ups1∆*) or the wild-type allele of *UPS1*, the main pathway for the CL accumulation appeared to change from the *UPS1*-dependent one to the FMM-dependent one. The differences of the decreases in the CL level among *tet-FMP30 ups2∆*, *tet-MDM31 ups2∆*, and *tet-MDM32 ups2∆* cells cultivated in the presence of Dox might be caused by the inability of complete shut-off of gene expression by Dox, and the difference in the minimal effective doses of Fmp30, Mdm31, and Mdm32. On the other hand, the FMM-dependent pathway seemed not to be responsible for the accumulation of the residual amount of CL in *ups1∆* cells with a normal mitochondrial PE level, because the CL levels in *tet-FMP30 ups1∆*, *tet-MDM31 ups1∆*, and *tet-MDM32 ups1∆* cells cultivated with Dox were similar to that in *ups1∆* cells (Figs 2a,b, 3b,c and 6b,c).

*FMP30* encodes a mitochondrial inner membrane protein with a large domain exposed to the intermembrane space, which exhibits a strong homology with mammalian NAPE-PLDs^27,29^. Mammalian NAPE-PLDs catalyze the hydrolysis of NAPE, resulting in the formation of PA and N-acylethanolamime. The substrates of Fmp30 remain to be established, but analyses of Fmp30 with point mutations have shown that the hydrolase activity of Fmp30 is essential for its function^27^. How does Fmp30 function in the FMM-dependent pathway for the CL accumulation? Given that one product of the enzymatic reaction catalyzed by Fmp30 is PA, a possible explanation for the enhancement is that Fmp30 provides PA used for CL biosynthesis in the MIM. In this regard, it is noteworthy that deletion of *FMP30* worsens the defects in cell growth and the CL level in yeast cells lacking the ERMES complex^27^, which is probably involved in PA transfer from the ER to mitochondria. The second explanation is that because PA is one of the cone shaped phospholipids, so-called “non-bilayer lipids”, the local synthesis of PA by Fmp30 induces membrane rearrangement, such as the formation of contact sites, which facilitates PA transfer from the MOM to the MIM, independently of Ups1-Mdm35. To address these possibilities, we are currently trying to clarify the substrates and products of the enzyme Fmp30.

*MDM31* and *MDM32* have been identified as genes required for the normal distribution and morphology of mitochondria^34^, and have been shown to encode homologous mitochondrial inner membrane proteins that have two membrane-spanning regions at the C-terminus and near the N-terminus, respectively, and a middle region exposed to the intermembrane space^35^, and form distinct protein complexes^35^. Deletion of *MDM31* or *MDM32* has been shown to decrease the mitochondrial CL level to about a half of that in wild-type mitochondria^22^. What might be the roles of Mdm31 and Mdm32 in the FMM-dependent pathway for the CL accumulation? Because Mdm31 and Mdm32 are MIM proteins with the same membrane topology, as described above, and exhibit 16.4% amino acid identity with each other^35^, these two proteins are supposed to have similar molecular functions. In the absence of Ups1, PA should be supplied to the MIM for CL synthesis in a Ups1-independent manner. In addition, deletion of *MDM31* or *MDM32* is synthetically lethal with deletion of the ERMES complex subunits involved in PA transfer from the ER to mitochondria^35^. Therefore, the simplest explanation for the roles of Mdm31 and Mdm32 in the CL accumulation is that these factors are involved in PA-supply to the MIM. The localization and topology of Mdm31 and Mdm32 are consistent with this explanation, but their amino acid sequences predict no domain structures and thus do not suggest their molecular functions. To address the molecular functions of Mdm31 and Mdm32, identification of the subunits of the Mdm31- or Mdm32-containing protein complexes^35^ would be an effective strategy, which is currently under investigation in this laboratory.

In conclusion, the present work shows that when the mitochondrial PE level is reduced to under the threshold level, Fmp30, Mdm31, and Mdm32 cooperatively function for the maintenance of a proper CL level even in the absence of Ups1-Mdm35-mediated PA-transfer to the MIM. This provides new insight into the CL metabolism and intramitochondrial transfer of phospholipids.

## Methods

### Yeast strains, genetic methods, media, and plasmids

The yeast strains used in this study are listed in Table S1. Yeast strains TKY705 (wild-type), TKY706 (wild-type α), TKY707 (*psd1∆*), and TKY709 (*psd1∆* α) were obtained by dissection of the asci in sporulated cultures of a diploid strain, TKY128^27^. Complete disruption, promoter replacement, and tagging of the yeast gene were accomplished by PCR-mediated gene replacement^36^ with a pair of primers and a template plasmid, as listed in Table S2.

Yeast cells were grown in YPAD (1% yeast extract, 2% peptone, 0.008% adenine, and 2% glucose, pH6.0). In some experiments, YPAD was supplemented with 10 µg/ml of a tetracycline analog, doxycycline (Dox) (Nakarai Chemicals), as indicated. SCAD medium (0.67% yeast nitrogen base without amino acids, 0.2% drop out mix, 0.008% adenine, and 2% glucose, pH6.0) without leucine was used for the selection of leucine-prototrophic transformants. Cells that have the *kanMX4* ^37^, *nat (natMX4* ^38^ and *natNT2* ^39^), and *hphNT1* ^39^ genes were selected with 200 µg/ml of G418 sulfate (Nakarai Chemicals), 100 µg/ml of clonNAT (Werner BioAgents), and 300 µg/ml of hygromycin B (Nakarai Chemicals), respectively. SCAD-MSG medium (0.17% yeast nitrogen base without amino acids and ammonium sulfate, 0.1% l-glutamic acid sodium salt, 0.2% drop out mix, 0.008% adenine, and 2% glucose, pH6.0) was used for random spore analysis^40^.

Plasmid pCM225 + S4 carrying the primer annealing sequence S4, which was used for promoter replacement with the tetO_7_ promoter^30^, was constructed as follows. A DNA fragment containing the tetO_7_ promoter sequence was amplified from pCM225^30^ using a forward primer containing a Xho1 site, 5′-AAGCTCCTCGAGTAATTCGC-3′, and a reverse primer containing a SfiI site and the S4 sequence, 5′-ATATGGCCGCATAGGCCCATCGATGAATTCTCTGTCGATAGGCCACTAGTGGATC-3′ (underlining, S4 sequence), digested with XhoI and SfiI, and then ligated with a large DNA fragment of pCM225 digested with XhoI and SfiI.

Plasmid pRS424-*FLAG-MDM31* encoding FLAG-tagged Mdm31 was constructed as follows. A DNA fragment encoding Mdm31 tagged with a FLAG epitope at near the N-terminus was constructed by PCR overlap extension recombination, using two pairs of primers (M13 rev primer (5′-CAGGAAACAGCTATGAC-3′) and FLAG-MDM31-R primer (5′-TGTCATCGTCATCCTTGTAATCCTCATTAGAATATGCTCTTAGC-3′), and FLAG-MDM31-F primer (5′-TACAAGGATGACGATGACAAGTCTAAAACTGGAAGGGATG-3′) and MDM31-R2 primer (5′-TCAATTGCGGTAGATCG-3′)), and a template plasmid, pRS424-*MDM31*, encoding Mdm31^28^ (Gift from Y. Tamura). The resulting fragment was digested with NotI and HpaI, and then ligated with a large DNA fragment of pRS424-MDM31 digested with NotI and HpaI.

Plasmid pRS424-*FLAG-MDM32* encoding FLAG-tagged Mdm32 was constructed as follows. A DNA fragment encoding Mdm32 tagged with a FLAG epitope at near the N-terminus was constructed by PCR overlap extension recombination, using two pairs of primers (M13 rev primer (5′-CAGGAAACAGCTATGAC-3′) and FLAG-MDM32-R primer (5′-TGTCATCGTCATCCTTGTAATCAGCCTTGGTAGTGAAC-3′), and FLAG-MDM32-F primer (5′-TACAAGGATGACGATGACAAGTCCAATATTGAGACTATTTTGC-3′) and MDM32-R2 primer (5′-CCGTGAAATCAAACTTCG-3′)), and a template plasmid, pRS424-*MDM32*, encoding Mdm32^28^ (Gift from Y. Tamura). The resulting fragment was digested with NotI and HpaI, and then ligated with a large DNA fragment of pRS424-MDM32 digested with NotI and HpaI.

### Analysis of cellular phospholipid compositions

Yeast cells were grown at 30 °C to saturation in YPAD supplemented with or without 10 µg/ml Dox. The cells were then diluted to an OD_600_ of 0.05 in YPD, and further incubated at 30 °C for 24 hours in the presence of 1 µCi/ml of [^32^P]Pi, supplemented with or without 10 µg/ml Dox. After incubation, cells were harvested, resuspended in 150 µl of 80% ethanol, heated at 95 °C for 15 min, mixed with 800 µl of chloroform/methanol (1:1, v/v), and then vortexed. 330 µl of 0.1 M HCl/0.1 M KCl was then added to the samples. The organic phase was separated by centrifugation at 3000 × *g* for 2 min. Samples containing equivalent radioactivity were collected and dried in a centrifugal evaporator and resuspended in chloroform/methanol (1:2, v/v). The samples were then subjected to TLC on a TLC plate (SILGUR-25-C/UV_254_; MACHEREY-NAGEL), which had been pretreated with 1.8% boric acid^41^, with the solvent system chloroform/ethanol/water/triethylamine (30:30:5:35, v/v). ^32^P-labeled phospholipids were detected and quantitated with an imaging analyzer, FLA-5000 (Fuji Photo Film) and MultiGauge software (Fuji Photo Film).

### Statistical analysis

The results of all quantitative experiments as means for independent experiments performed multiple times as indicated. The statistical significance of mean differences was assessed by means of two-tailed Student’s t-test.

### Immunoprecipitation

2 mg of yeast mitochondria was solubilized with lysis buffer (20 mM HEPES-KOH (pH 7.4), 100 mM KCl, 10% glycerol, 1% digitonin, and complete mini EDTA-free (Roche)). The lysates were incubated with anti-FLAG agarose beads (Sigma-Aldrich) at 4 °C for 2 h. After washing the beads with lysis buffer three times, immunoprecipitates were eluted with 2% SDS. The eluates were then analyzed by Western blotting using antibodies against HA (Santa Cruz), FLAG M2 (Sigma-Aldrich), and Tim23 (A gift from T. Endo, Kyoto Sangyo University, Kyoto, Japan).

## Electronic supplementary material

Supplementary information
