# Supplementary material for: Cooperative function of Fmp30, Mdm31, and Mdm32 in Ups1-independent cardiolipin accumulation in the yeast Saccharomyces cerevisiae
Source: Sci Rep. 2017 Nov 27;7:16447. doi: 10.1038/s41598-017-16661-2 (PMC5703896; doi:10.1038/s41598-017-16661-2)
Supplement: Supplementary file 1 — Supplementary information [file 41598_2017_16661_MOESM1_ESM.pdf]

Cooperative function of Fmp30, Mdm31, and Mdm32 in Ups1-independent cardiolipin accumulation in the yeast *Saccharomyces cerevisiae*

Non Miyata[1], Naoto Goda[1], Keiji Matsuo, Takeshi Hoketsu, and Osamu Kuge[*]

Department of Chemistry, Faculty of Science, Kyushu University, Fukuoka 819-0395, Japan
[*]Correspondence to Osamu Kuge, kuge@chem.kyushu-univ.jp
[1]These authors contributed equally to this work

Supplementary Information

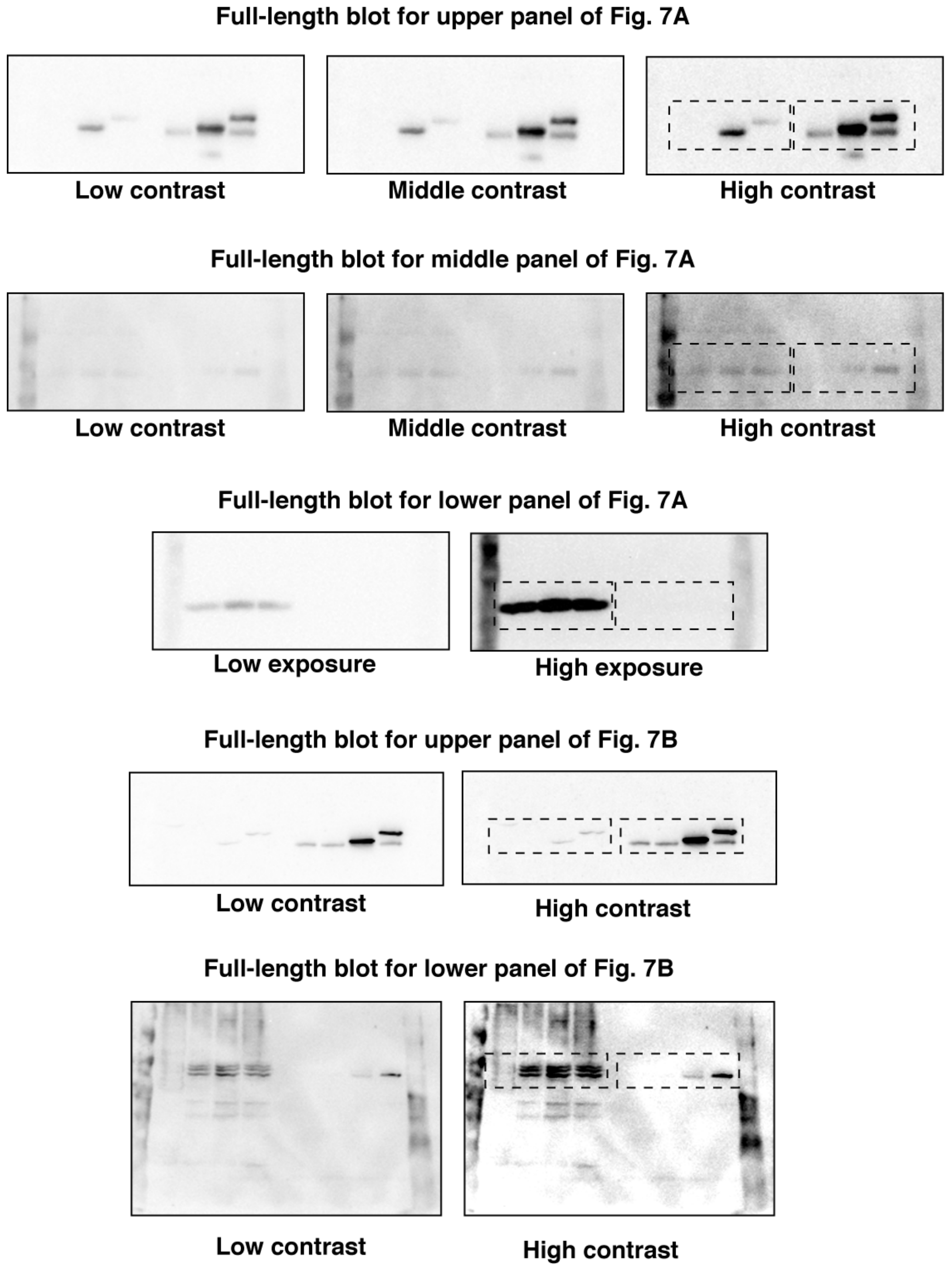

Supplementary Figure S1. Multiple exposures of full-length blots for Fig. 7. Images surrounded with dashed lines were included in Fig. 7.

Table S1. Yeast strains used in this study.

Strain description (Strain name)	Genotype	Source
Wild Type (TKY705)	*MAT*a *ura3-52 trp1Δ2 leu2-3_112 his3-11 ade2-1* $CAN1^{WT}$ (BMA64-1A, $CAN1^{WT}$) (Genetic background: W303)	This study
ups1Δ (OKY7130)	TKY705, *ups1Δ::hphNT1*	This study
ups2Δ (OKY7063)	TKY705, *ups2Δ::CgLEU2*	Miyata et al.[1)]
psd1Δ (TKY707)	TKY705, *psd1Δ::kanMX4*	This study
cho1Δ (OKY7037)	TKY705, *cho1Δ::kanMX4*	This study
ups1Δ ups2Δ (OKY7136)	TKY705, *ups1Δ::hphNT1 ups2Δ::CgLEU2*	This study
ups1Δ psd1Δ (OKY7132)	TKY705, *ups1Δ::hphNT1 psd1Δ::kanMX4*	This study
ups1Δ cho1Δ (OKY7131)	TKY705, *ups1Δ::hphNT1 cho1Δ::kanMX4*	This study
PSD1↑CHO1↑ (OKY7153)	TKY705, *GPDpr-PSD1::natNT2 GPD-pr-CHO1::kanMX4*	This study
ups1Δups2ΔPSD1↑CHO↑ (OKY7143)	TKY705, *ups1Δ::hphNT1 ups2Δ::CgLEU2 GPDpr-PSD1::natNT2 GPD-pr-CHO1::kanMX4*	This study
tet-FMP30 (OKY7139)	TKY705, $tetO_7$*-FMP30::kanMX4*	This study
tet-FMP30 ups1Δ (OKY7140)	TKY705, $tetO_7$*-FMP30::kanMX4 ups1Δ::hphNT1*	This study
tet-FMP30 ups2Δ (OKY7141)	TKY705, $tetO_7$*-FMP30::kanMX4 ups2Δ::CgLEU2*	This study
tet-FMP30 psd1Δ (OKY7157)	TKY705, $tetO_7$*-FMP30::kanMX4 psd1Δ::natMX4*	This study
tet-FMP30 cho1Δ (OKY7156)	TKY705, $tetO_7$*-FMP30::kanMX4 cho1Δ::natMX4*	This study
tet-FMP30 ups1Δ ups2Δ (OKY7142)	TKY705, $tetO_7$*-FMP30::kanMX4 ups1Δ::hphNT1 ups2Δ::CgLEU2*	This study
tet-FMP30 ups1Δ psd1Δ (OKY7160)	TKY705, $tetO_7$*-FMP30::kanMX4 ups1Δ::hphNT1 psd1Δ::natMX4*	This study
tet-FMP30 ups1Δ cho1Δ (OKY7159)	TKY705, $tetO_7$*-FMP30::kanMX4 ups1Δ::hphNT1 cho1Δ::natMX4*	This study
mdm31Δ a (OKY7016)	TKY705, *mdm31Δ::hphNT1*	This study
mdm32Δ a (OKY7017)	TKY705, *mdm32Δ::natMX4*	This study
tet-MDM31 (OKY7171)	TKY705, $tetO_7$*-MDM31::kanMX4*	This study
tet-MDM32 (OKY7172)	TKY705, $tetO_7$*-MDM32::kanMX4*	This study
tet-MDM31 ups1Δ (OKY7174)	TKY705, $tetO_7$*-MDM31::kanMX4 ups1Δ::hphNT1*	This study
tet-MDM32 ups1Δ (OKY7175)	TKY705, $tetO_7$*-MDM32::kanMX4 ups1Δ::hphNT1*	This study
tet-MDM31 ups2Δ (OKY7176)	TKY705, $tetO_7$*-MDM31::kanMX4 ups2Δ::CgLEU2*	This study
tet-MDM32 ups2Δ (OKY7177)	TKY705, $tetO_7$*-MDM32::kanMX4 ups2Δ::CgLEU2*	This study
tet-MDM31 ups1Δ ups2Δ (OKY7178)	TKY705, $tetO_7$*-MDM31::kanMX4 ups1Δ::hphNT1 ups2Δ::CgLEU2*	This study
tet-MDM32 ups1Δ ups2Δ (OKY7179)	TKY705, $tetO_7$*-MDM32::kanMX4 ups1Δ::hphNT1 ups2Δ::CgLEU2*	This study
FMP30-3xHA (OKY7137)	TKY705, *FMP30-3xHA::kanMX4*	This study
GPDpr-FMP30-3xHA (OKY7197)	TKY705, *FMP30-3xHA::kanMX4 GPDpr-FMP30::natNT2*	Thisstudy
Wild Type α (TKY706)	*MAT*α *ura3-52 trp1Δ2 leu2-3_112 his3-11 ade2-1 can1Δ::STE2pr-Sp_his5* (BMA64-1B, *can1Δ::STE2pr-Sp_his5*) (Genetic background: W303)	This study
psd1Δ α (TKY709)	TKY706, *psd1Δ::kanMX4*	This study

1) Miyata, N., Watanabe, Y., Tamura, Y., Endo, T. & Kuge, O. Phosphatidylserine transport by Ups2-Mdm35 in respiration-active mitochondria. *J Cell Biol* **214**, 77-88 (2016).

Table S2. PCR templates and primers used for gene manipulation.

Gene	Template plasmid (Source)	Primers (5' to 3')
*ups1*Δ*::hphNT1*	pFA6a-*hph-NT1*[1)] (EUROSCARF)	1: TCTGGCTTCTGAGACGGCGGTAAGATATCCTTAAGAGTTGCAATGCGTACGCTGCAGGTCGAC<br>2: CTCGCCCATGGTGATATCTTTAAAGATCTTTAAATGGGAACATCAATCGATGAATTCGAGCTCG
*ups2*Δ*::CgLEU2*	pCg*LEU2-NT1*[2)] (This laboratory)	1: TCAGACTAAGATAAAATAATCGAGAATAATTAAAAGACGATAATGCGTACGCTGCAGGTCGAC<br>2: AAGTAGTATGCAGTGCCATGCGGGATCAAGGAATTTGTATCTCTAATCGATGAATTCGAGCTCG
*cho1*Δ*::kanMX4*	pFA6a-*kan-MX4*[3)] (EUROSCARF)	1: TTGATTCAATCAAAAAACAAAAATAAAACTATATATTAAAAAATGACATGGAGGCCCAGAATACCCT<br>2: TATGTACAAATTTTTTTTGACGCCAGGCATGAACAAAAACTACTACAGTATAGCGACCAGCATTCAC
*psd1*Δ*::natMX4*	p4339[4)] (Gift from C. Boone)	1: TTGGTCGTTATTTTTTGAAGAAGAAGGAAAAGCAAAGCCAGCATGCGTACGCTGCAGGTCGAC<br>2: TATATACAGCAAAATAAATGCTAACTTTACATATGATTGCTTTCAATCGATGAATTCGAGCTCG
*cho1*Δ*::natMX4*	p4339[4)] (Gift from C. Boone)	1: TTGATTCAATCAAAAAACAAAAATAAAACTATATATTAAAAAATGCGTACGCTGCAGGTCGAC<br>2: TATGTACAAATTTTTTTTGACGCCAGGCATGAACAAAAACTACTAATCGATGAATTCGAGCTCG
GPDpr-PSD1::natNT2	pYM-N15[1)] (EUROSCARF)	1: TTGGTCGTTATTTTTTGAAGAAGAAGGAAAAGCAAAGCCAGCATGCGTACGCTGCAGGTCGAC<br>2: GAGCGTCCTCCCTTGCGCCAAGGCGTTCTTAACTGGCATAATTGACATCGATGAATTCTCTGTCG
GPDpr-CHO1::kanMX4	pYM-N14[1)] (EUROSCARF)	1: TTGATTCAATCAAAAAACAAAAATAAAACTATATATTAAAAAATGCGTACGCTGCAGGTCGAC<br>2: CGTGTGTGGGAATTCTTGAGGTGCGAAATCTTCATCTGATTCAACCATCGATGAATTCTCTGTCG
$tetO_7$*-FMP30::kanMX4*	pCM225 +S4 (This study)	1: AGTAGCACTCCAATTACTGAAGGATTGCATGCCTGCTTGTGTTGCAAATGCGTACGCTGCAGGTCGAC<br>2: AGTCTCCTTTGGAGAAGAAGCCTCATCTGAACATGGCAGGTAACAAAATTCATCGATGAATTCTCTGTCG
*mdm31*Δ*::hphNT1*	pFA6a-*hph-NT1*[1)] (EUROSCARF)	1: ATATATGTAAATTCGGACGGGAAAGCTGGCAGTCTACTATATGCGTACGCTGCAGGTCGAC<br>2: TAAATGCATATGTACAGAAAGTGACAGGAGTAGGTATTCCTCAATCGATGAATTCGAGCTCG
*mdm32*Δ*::natMX4*	p4339[4)] (Gift from C. Boone)	1: AAAGGCACATACAGATTATCAGGTGGTGGGAGGTTTATAAAATTCATGACATGGAGGCCCAGAATACCCT<br>2: AAATGAAATGCAAGGAAGTTCAGTTTGATATTATATAAGCAATAGTCACAGTATAGCGACCAGCATTCAC
$tetO_7$*-MDM31::kanMX4*	pCM225 +S4 (This study)	1: ATATATGTAAATTCGGACGGGAAAGCTGGCAGTCTACTATATGCGTACGCTGCAGGTCGAC<br>2: GCTACAGAAAACTGCCGGGGGCTCCTTAGAAAAGGCCTGGTAAAAAGGGACATCGATGAATTCTCTGTCG
$tetO_7$*-MDM32::kanMX4*	pCM225 +S4 (This study)	1: CAAAAGGCACATACAGATTATCAGGTGGTGGGAGGTTTATAAAATTCATGCGTACGCTGCAGGTCGAC<br>2: ATTGGAAGCAGTGGCCGCTTTATTGTAGGGACCCGTAAACGTGTGATTAGCATCGATGAATTCTCTGTCG
FMP30-3xHA::kanMX4	pYM1[5)] (Gift from E. Schiebel)	1: TTAAAGACCGCTGCTACTGTCCAGAATTGGGCAAGACAGAATGCTTCGACCGTACGCTGCAGGTCGAC<br>2: TTATCGCTATTTACAGGGACGCTGCAGCTTAAATATTAGCGCCTAAGTTAATCGATGAATTCGAGCTCG
GPDpr-FMP30::nanNT2	pYM-N15[1)] (EUROSCARF)	1: AGTAGCACTCCAATTACTGAAGGATTGCATGCCTGCTTGTGTTGCAAATGCGTACGCTGCAGGTCGAC<br>2: AGTCTCCTTTGGAGAAGAAGCCTCATCTGAACATGGCAGGTAACAAAATTCATCGATGAATTCTCTGTCG

1) Janke, C. *et al.* A versatile toolbox for PCR-based tagging of yeast genes: new fluorescent proteins, more markers and promoter substitution cassettes. *Yeast* **21**, 947-962 (2004).

2) Miyata, N., Watanabe, Y., Tamura, Y., Endo, T. & Kuge, O. Phosphatidylserine transport by Ups2-Mdm35 in respiration-active mitochondria. *J Cell Biol* **214**, 77-88 (2016).

3) Wach, A., Brachat, A., Pohlmann, R. & Philippsen, P. New heterologous modules for classical or PCR-based gene disruptions in *Saccharomyces cerevisiae*. *Yeast* **10**, 1793-1808 (1994).

4) Tong, A. H. & Boone, C. Synthetic genetic array analysis in Saccharomyces cerevisiae. *Methods Mol Biol* **313**, 171-192 (2006).

5) Knop, M. *et al.* Epitope tagging of yeast genes using a PCR-based strategy: More tags and improved practical routines. *Yeast* **15**, 963-972 (1999)

Table S3. CL and PE levels in, and growth rate on YPAD plate of various yeast mutants

**Strain**	**CL level (%)**	**PE level (%)**	**Growth**
Wild type	**100**	**100**	++++
ups1Δ	23	95	++
ups1Δups2Δ	75	71	+++
ups1Δpsd1Δ	52	57	+++
ups1Δcho1Δ	58	27	+++
ups1Δups2Δ + 2OE	13	147	+
ups2Δ	92	68	++++
psd1Δ	72	57	++++
cho1Δ	63	28	+++
2OE	73	130	++++
tet-FMP30 -Dox	97	97	++++
tet-FMP30 +Dox	91	96	++++
tet-FMP30 ups1Δ -Dox	24	76	++
tet-FMP30 ups1Δ +Dox	19	78	+
tet-FMP30 ups1Δups2Δ -Dox	82	62	+++
tet-FMP30 ups1Δups2Δ +Dox	12	52	+
tet-FMP30 ups1Δpsd1Δ -Dox	61	52	+++
tet-FMP30 ups1Δpsd1Δ +Dox	8	43	+
tet-FMP30 ups1Δcho1Δ -Dox	55	22	+++
tet-FMP30 ups1Δcho1Δ +Dox	13	24	+
tet-FMP30 ups2Δ -Dox	82	67	Not shown
tet-FMP30 ups2Δ +Dox	50	72	Not shown
tet-FMP30 psd1Δ -Dox	62	57	Not shown
tet-FMP30 psd1Δ +Dox	25	60	Not shown
tet-FMP30 cho1Δ -Dox	74	30	Not shown
tet-FMP30 cho1Δ +Dox	44	38	Not shown
tet-MDM31 -Dox	93	99	++++
tet-MDM31 +Dox	87	94	++++
tet-MDM31 ups1Δ -Dox	47	100	+++
tet-MDM31 ups1Δ +Dox	19	84	++
tet-MDM31 ups2Δ -Dox	80	65	++++
tet-MDM31 ups2Δ +Dox	27	70	+++
tet-MDM31 ups1Δups2Δ -Dox	80	62	++++
tet-MDM31 ups1Δups2Δ +Dox	12	60	++
tet-MDM32 -Dox	82	89	++++
tet-MDM32 +Dox	69	91	++++
tet-MDM32 ups1Δ -Dox	31	89	++
tet-MDM32 ups1Δ +Dox	8	68	+
tet-MDM32 ups2Δ -Dox	75	63	++++
tet-MDM32 ups2Δ +Dox	15	61	++
tet-MDM32 ups1Δups2Δ -Dox	59	67	+++
tet-MDM32 ups1Δups2Δ +Dox	3	50	+
